# Randomized, multicenter study: on-demand versus continuous maintenance treatment with esomeprazole in patients with non-erosive gastroesophageal reflux disease

**DOI:** 10.1186/s12876-016-0448-x

**Published:** 2016-04-14

**Authors:** Ekkehard Bayerdörffer, Marc-Andre Bigard, Werner Weiss, Fermín Mearin, Luis Rodrigo, Juan Enrique Dominguez Muñoz, Hennie Grundling, Tore Persson, Lars-Erik Svedberg, Nanna Keeling, Stefan Eklund

**Affiliations:** Department of Internal Medicine, Lohr Health Centre, Lohr, Germany; Gastroenterology Unit, University Hospital, Vandoeuvre les Nancy, France; 4th Medical Department, Hospital Rudolfstiftung, Vienna, Austria; Gastroenterology Service, Centro Médico Teknon, Barcelona, Spain; Gastroenterology Service, Hospital Central de Asturias, Oviedo, Spain; Department of Gastroenterology, University Hospital, Santiago de Compostela, Spain; Department of Internal Medicine, Universitas Hospital, Bloemfontein, South Africa; AstraZeneca R&D, Gothenburg, Sweden

**Keywords:** Discontinuation, Esomeprazole, Gastroesophageal reflux disease, Heartburn, Non-erosive reflux disease, On-demand

## Abstract

**Background:**

Most patients with gastroesophageal reflux disease experience symptomatic relapse after stopping acid-suppressive medication. The aim of this study was to compare willingness to continue treatment with esomeprazole on-demand versus continuous maintenance therapy for symptom control in patients with non-erosive reflux disease (NERD) after 6 months.

**Methods:**

This multicenter, open-label, randomized, parallel-group study enrolled adults with NERD who were heartburn-free after 4 weeks’ treatment with esomeprazole 20 mg daily. Patients received esomeprazole 20 mg daily continuously or on-demand for 6 months. The primary variable was discontinuation due to unsatisfactory treatment. On-demand treatment was considered non-inferior if the upper limit of the one-sided 95 % confidence interval (CI) for the difference between treatments was <10 %.

**Results:**

Of 877 patients enrolled, 598 were randomized to maintenance treatment (continuous: *n* = 297; on-demand: *n* = 301). Discontinuation due to unsatisfactory treatment was 6.3 % for on-demand and 9.8 % for continuous treatment (difference −3.5 % [90 % CI: −7.1 %, 0.2 %]). In total, 82.1 and 86.2 % of patients taking on-demand and continuous therapy, respectively, were satisfied with the treatment of heartburn and regurgitation symptoms, a secondary variable (*P* = NS). Mean study drug consumption was 0.41 and 0.91 tablets/day, respectively. Overall, 5 % of the on-demand group developed reflux esophagitis versus none in the continuous group (*P* < 0.0001). The Gastrointestinal Symptom Rating Scale Reflux dimension was also improved for continuous versus on-demand treatment. Esomeprazole was well tolerated.

**Conclusions:**

In terms of willingness to continue treatment, on-demand treatment with esomeprazole 20 mg was non-inferior to continuous maintenance treatment and reduced medication usage in patients with NERD who had achieved symptom control with initial esomeprazole treatment.

**Trial registration:**

ClinicalTrials.gov identifier (NCT number): NCT02670642; Date of registration: December 2015.

## Background

Gastroesophageal reflux disease (GERD) is a chronic condition characterized by a range of symptoms, the most important of which are heartburn and regurgitation [1, 2]. Population-based studies have shown that these symptoms affect as many as 20 % of people on a weekly basis [3]. Although GERD is associated with reflux esophagitis, which can be detected and confirmed by esophageal endoscopy, as many as 70 % of patients with GERD display no such endoscopic findings and are thus termed as having endoscopy-negative or non-erosive reflux disease (NERD) [4]. Despite the absence of esophagitis, many of these patients experience a significant impairment in their health-related quality of life (HRQoL), similar to that experienced by patients with reflux esophagitis [4–6].

As the severity of symptoms generally correlates with esophageal acid exposure [7, 8], the most effective treatment for GERD, including NERD, is acid-suppressive therapy with a proton pump inhibitor (PPI) [9]. It has been shown that continuous maintenance treatment with the PPI esomeprazole (20 mg once daily) provides more effective acid suppression and maintained intragastric pH >4 for a greater period of time than maintenance omeprazole, lansoprazole, pantoprazole or rabeprazole in patients with GERD [10, 11]. In addition, a study by Talley et al. showed that, in patients with NERD, 6 months’ on-demand treatment with esomeprazole 20 mg controlled symptoms in 92 % of patients who were using only 33 % of the medication needed for daily continuous treatment [12]. In our study, we compared the efficacy of on-demand versus continuous esomeprazole maintenance treatment in patients with NERD (who had complete resolution of heartburn symptoms following initial treatment with esomeprazole) in terms of the willingness of patients to continue therapy.

## Methods

### Patients

Patients presenting to their general practitioner or gastrointestinal (GI) specialist with symptoms suggestive of GERD and with heartburn as their predominant symptom for longer than 6 months were considered for study entry. In addition, patients were included if they met the following inclusion criteria: age ≥18 years (Austria, ≥19 years); heartburn occurring for ≥4 days during the last 7 days before endoscopy; or, if PPI therapy had been started within the last 7 days before endoscopy, heartburn occurring for ≥4 days during the last 7 days before the start of PPI treatment.

Patients were excluded if they had any of the following: significant GI disorders or other disorders likely to affect the outcome of the study; gastroduodenal ulcers within the previous 2 years; previous esophageal, gastric or duodenal surgery (except closure and oversewing of an ulcer); irritable bowel syndrome; PPI use for either ≥10 of the 28 days before endoscopy or ≥5 of the 7 days before endoscopy; use of concomitant therapy likely to affect the outcome of the trial; pregnancy or lactation; childbearing potential unless taking effective contraception; and alcohol or drug abuse.

### Study design

**Fig. 1:**
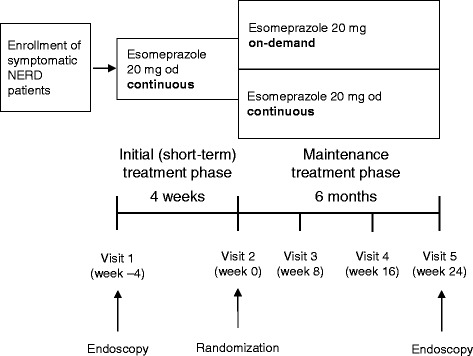
Study design. Abbreviations: NERD, non-erosive reflux disease (without mucosal breaks on pre-treatment endoscopy); od, once daily

This was a multicenter, open-label, randomized, parallel-group study (Fig. 1). Patients were enrolled at 61 centers in Austria, France, Germany, South Africa and Spain between August 2001 and April 2002. The study was performed in accordance with the most recent amendment to the Declaration of Helsinki and complied with Good Clinical Practice. At each participating center, a local independent ethics committee or institutional review board approved the final study protocol. Signed informed consent was obtained from all patients before conducting any specific procedures for the study.

At visit 1 (week −4), a medical history was taken, and physical examination and endoscopy were performed. Endoscopy was performed again at visit 2 (week 0), and patients with reflux esophagitis detected at either visit 1 or 2 were excluded from randomization. Reflux esophagitis was defined as endoscopy-confirmed mucosal breaks [13]. *Helicobacter pylori* status was assessed at visit 1 on two antral and two corpus biopsy specimens. Specimens were evaluated by one central pathologist according to the criteria of the Sydney classification [14]. Patients with positive *H. pylori* status did not receive any eradication treatment during the study period.

All eligible patients underwent an initial (short-term) treatment period of 4 weeks with esomeprazole 20 mg tablets once daily (administered as 22.3 mg esomeprazole magnesium trihydrate). Severity of symptoms (heartburn, acid regurgitation, dysphagia and epigastric pain) was assessed as none, mild, moderate or severe at visits 1 (week −4) and 2 (week 0) using standard questions posed by the investigator. The frequency of heartburn was also reported. Only patients who were free from heartburn at visit 2 (defined as 7 symptom-free days in the last week of the short-term treatment phase; i.e., complete resolution of symptoms) were randomized sequentially (1:1) to one of two treatment groups for a 6-month maintenance treatment phase. Patients in the on-demand treatment group received esomeprazole 20 mg tablets (up to a maximum of once daily), taken as needed to adequately control their reflux symptoms; treatment could be taken to prevent symptoms, to soothe symptoms, or both. Specific circumstances prompting each on-demand use of esomeprazole were not recorded, although at the end of the 6-month treatment period patients were asked whether they had taken their medicine to soothe or prevent symptoms, or both. Patients in the continuous treatment group received esomeprazole 20 mg tablets once daily continuously (Fig. 1). Randomization was performed using a computer program at AstraZeneca in balanced blocks using a blocking size of 2.

Other PPIs and H_2_-receptor antagonists were not permitted during treatment. Antacids could only be taken between initial endoscopy and first administration of study drug.

### Study measurements and variables

The primary variable was the proportion of patients discontinuing the study as a result of unsatisfactory treatment. At clinical visits 2 to 5 (weeks 0, 8, 16 and 24 of the maintenance treatment phase) the investigator confirmed with the patient if he/she wished to continue with the treatment and, if not, the date and reasons for discontinuation were recorded. Following discontinuation of esomeprazole, patients were treated at the discretion of their investigator with medicines that were available in their country.

Secondary variables included the reasons given for treatment discontinuation, including: dissatisfaction with symptom control, the method of administration (on-demand or continuous) or taste/size of the pill; adverse events (AEs); protocol non-compliance; inclusion criteria not fulfilled (retrospective); patient lost to follow-up; improvement/recovery as evaluated by the investigator; or other reason specified by the investigator.

Treatment satisfaction was evaluated using a standardized questionnaire completed by patients at visits 2 to 5 (weeks 0, 8, 16 and 24 of the maintenance treatment phase), or at premature discontinuation. The questionnaire comprised three questions: “How satisfied or dissatisfied are you with the effect of the drug?”; “How satisfied or dissatisfied are you with the way of taking the drug?”; and “Overall, how satisfied or dissatisfied are you with the way of treating your heartburn and regurgitation symptoms?”. Patients were asked to give their answers as “completely satisfied”, “quite satisfied”, “neither satisfied nor dissatisfied”, “quite dissatisfied” or “completely dissatisfied”. For the purpose of this analysis, “satisfied” was defined as the sum of the upper two ratings (“completely satisfied” and “quite satisfied”).

The intake of study medication was registered using the MEMS® device, which utilizes a microelectronic recorder recessed in the cap of a drug container (Medical Event Monitoring System, Aardex, Zug, Switzerland). At each opening and closure of the container, the date and time of day was automatically recorded. This information was analyzed at the end of the study.

The evaluation of patient-reported outcomes focused on reflux symptoms and the impact on patients’ quality of daily life. Symptom assessments were carried out using a standardized patient-reported outcomes questionnaire, the Gastrointestinal Symptom Rating Scale (GSRS), which has been validated in symptomatic GERD [15]. The GSRS consists of 15 GI symptoms grouped into 5 dimensions. Each dimension is scored on a 7-point scale, with a lower score indicating a lower perceived symptom severity. HRQoL assessments were made using the Quality of Life in Reflux and Dyspepsia (QOLRAD) instrument [16, 17], which was specifically developed for patients with symptoms of reflux and dyspepsia. The QOLRAD questionnaire consists of 25 items grouped into 5 dimensions representing different aspects of the daily life of patients with GERD. The questionnaire uses a similar 7-point scoring system to the GSRS; however, a lower score indicates a more severe impact on daily functioning. The GSRS and QOLRAD questionnaires were completed by the patients prior to treatment starting at visit 1 and at all subsequent visits. The changes in these parameters from start of the short-term and maintenance treatment phases to the end of the study were assessed and compared between the two treatments.

Data for all patient-reported outcomes were collected electronically by patients using a hand-held computer (Newton MessagePad 130/2000; Apple Computer, Inc., Cupertino, CA, USA). Use of an electronic questionnaire, rather than a paper-based format, was selected in view of the associated benefits of completeness of data, speed of data flow and ease of data handling [18].

#### Safety and tolerability

AE and serious AE (SAE) assessments were recorded throughout the short-term and maintenance treatment phases of the study (visits 1–5). Blood samples for laboratory screening were taken before administration of the study drug (at visit 1) and at study end point or premature discontinuation.

#### Endoscopic assessment

An endoscopic assessment of the esophageal mucosa was performed at visits 1 (or 7 days before) and 5 (end of study), and for all patients who left the study prematurely. The Los Angeles (LA) classification system [19] was used to grade the appearance of mucosal breaks.

### Statistical analysis

The proportion of patients who were free of heartburn at the end of the initial 4-week treatment phase was calculated for the safety population, which comprised all patients who took at least one dose of study drug and for whom post-dose information was available. All patients randomized to maintenance treatment were included in the intention-to-treat (ITT) analysis set, and the primary variable was analyzed for these patients. ITT data were analyzed using the last-visit-carried-forward method for those patients who discontinued prematurely, and all patients who discontinued were regarded as having discontinued due to unsatisfactory treatment. ITT patients were included in the per-protocol (PP) analysis set if they were assessed for the primary variable. Patients were excluded from the PP population due to: violation of the inclusion/exclusion criteria; treatment with disallowed concomitant medication; compliance less than 75 % in the treatment period; or other deviations from study procedures.

Estimates and two-sided 90 % confidence intervals (CIs) were calculated for the difference between the on-demand and continuous treatment groups in the proportion of patients who discontinued prematurely because of dissatisfaction with study treatment. On-demand treatment was considered to be non-inferior to continuous treatment if the one-sided 95 % CI for the difference between treatments, shown by the upper bound of the two-sided 90 % CI, was less than 10 %, as pre-specified in the clinical study protocol [12, 20]. The difference between the groups in the proportion of patients who discontinued (overall and due to an AE) was compared using Chi-square tests with Yates’ correction.

The secondary variables were analyzed for the ITT population only. The reasons for discontinuation from treatment and drug usage (date and time recorded by the MEMS® device) were recorded descriptively.

Results from the treatment satisfaction questions were presented using estimates for the proportion of satisfied patients for the two treatment groups. In addition, estimates and two-sided 95 % CIs (based on normal approximation) for the treatment difference in the proportion of satisfied patients were assessed.

For patient-reported changes in symptoms and HRQoL, the changes from baseline visit 1 (week −4) to end point and from visit 2 (week 0) to end point were calculated for the separate dimensions of GSRS and QOLRAD with two-sided 95 % CIs. The differences between the two treatment groups were compared for each dimension using analysis of variance.

Safety data were presented for the safety population (as defined above).

#### Determination of sample size

Sample size was calculated from numbers needed to show that on-demand treatment was non-inferior to continuous treatment based on the proportion of patients who would discontinue the study prematurely because of unsatisfactory therapy. As outlined above, on-demand treatment was defined as non-inferior to continuous treatment if the upper bound of the two-sided 90 % CI for the difference in proportions (on-demand minus continuous) was less than 10 %.

With a sample size of 275 patients in each treatment group, a one-sided test of proportions at the 5 % level had 90 % power to reject the hypothesis that on-demand treatment is inferior to continuous treatment. With an estimated 10 % non-evaluable patients and assuming that only 60 % of the patients would be randomized (conservative estimates based on data from previous studies), it was calculated that 1020 patients would be required in the initial treatment phase to yield sufficient power.

## Results

### Patient disposition and demographics

**Fig. 2:**
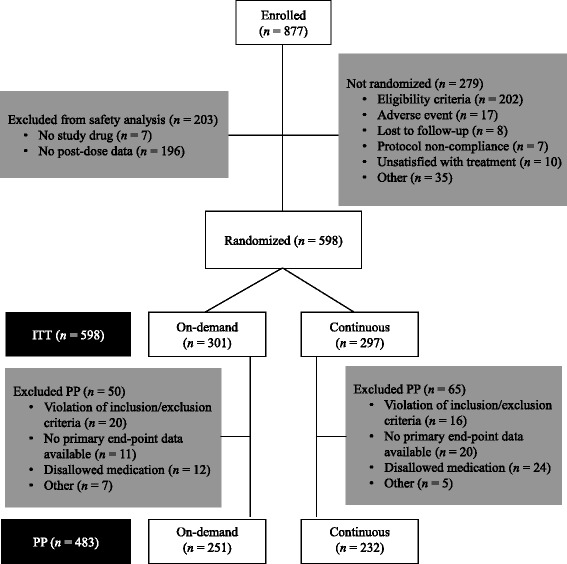
Flow diagram of patients’ disposition through the stages of the study. Abbreviations: ITT, intention-to-treat; PP, per-protocol

Patients were enrolled at 6 centers in Austria (*n* = 65), 27 in France (*n* = 211), 14 in Germany (*n* = 182), 9 in South Africa (*n* = 289) and 5 in Spain (*n* = 130). Patient disposition through each stage of the study is shown in Fig. 2. In total, 877 patients were enrolled at visit 1 and 598 (68 %) eligible patients were randomized to maintenance treatment at visit 2. The safety population comprised 674 patients during the short-term treatment phase and 595 patients (on-demand, *n* = 301; continuous, *n* = 294) during the maintenance treatment phase. During the 4-week short-term treatment period, a high proportion of patients (619/674, 92 %) were free of heartburn (investigator-assessed); this was one of the eligibility criteria for the maintenance treatment phase of the study.

All 598 patients randomized to the maintenance treatment phase were included in the ITT analysis and 483 of these fulfilled the criteria for inclusion in the PP analysis. Of the 279 patients who were enrolled into the study but not randomized, 202 did not fulfil the eligibility criteria (most because of the presence of esophagitis at endoscopic examination at either the enrollment or randomization visit), including 44 patients who had heartburn for at least one day in the week prior to randomization. Of the 115 patients excluded from the PP analysis, 36 had received disallowed medication during maintenance treatment. Overall, the maintenance treatment groups were well balanced with respect to demographic and baseline characteristics (Table 1).

**Table 1:** Baseline demographics (intention-to-treat population)

Characteristic	On-demand (*n* = 301)	Continuous (*n* = 297)
Men, *n* (%)	122 (40.5)	130 (43.8)
Women, *n* (%)	179 (59.5)	167 (56.2)
Ethnicity, *n* (%)		
White	259 (86.0)	255 (85.9)
Black	14 (4.7)	10 (3.4)
Asian	1 (0.3)	4 (1.3)
Other^a^	27 (9.0)	28 (9.4)
Age (y), mean ± SD	48.2 ± 13.6	47.6 ± 15.1
Height (cm), mean ± SD	167.0 ± 9.1	167.0 ± 9.9
Weight (kg), mean ± SD	75.1 ± 14.3	75.8 ± 14.7
Hiatal hernia, *n* (%)	96 (31.9)	108 (36.4)
Days with heartburn, *n* (%)		
4 d	26 (8.6)	31 (10.4)
5 d	55 (18.3)	46 (15.5)
6 d	46 (15.3)	42 (14.1)
7 d	174 (57.8)	178 (59.9)
Severity of heartburn, *n* (%)		
Mild	21 (7.0)	19 (6.4)
Moderate	165 (54.8)	153 (51.5)
Severe	115 (38.2)	125 (42.1)
Severity of acid regurgitation, *n* (%)		
None	82 (27.2)	63 (21.2)
Mild	68 (22.6)	76 (25.6)
Moderate	95 (31.6)	95 (32.0)
Severe	56 (18.6)	63 (21.2)
Severity of epigastric pain, *n* (%)		
None	133 (44.2)	129 (43.4)
Mild	96 (31.9)	81 (27.3)
Moderate	54 (17.9)	64 (21.5)
Severe	18 (6.0)	23 (7.7)
Severity of dysphagia, *n* (%)		
None	265 (85.0)	255 (85.9)
Mild	28 (9.3)	22 (7.4)
Moderate	14 (4.7)	14 (4.7)
Severe	3 (1.0)	6 (2.0)
*Helicobacter pylori*-positive, *n* (%)	125 (41.5)	130 (43.8)

^a^Including patients of mixed ethnicity

*Abbreviation*: *SD* standard deviation

When viewed by country, the demographic data were similar in terms of age and gender. Austria, Germany and France showed similar proportions of patients who were *H. pylori*-positive (26.7, 28.8 and 34.2 %, respectively). However, the proportions for South Africa and Spain were noticeably higher (63.4 and 54.7 %, respectively).

### Primary variable

In total, 48 (8.0 %) patients discontinued from the study prematurely, 19 (6.3 %) in the on-demand treatment group and 29 (9.8 %) in the continuous treatment group (*P* = 0.15). In the ITT analysis, all premature discontinuations were regarded as being due to unsatisfactory treatment. The distribution of reasons for discontinuation was similar in the two treatment groups (Table 2). Five patients (on-demand, *n* = 3; continuous, *n* = 2) actively reported that they discontinued the study because of unsatisfactory control of symptoms and no patient in either group discontinued because of dissatisfaction with the way of taking the drug or the taste/size of the tablet.

**Table 2:** Reasons for discontinuation due to unsatisfactory treatment (intention-to-treat population)

	Patients [*n* (%)]	
	On-demand (*n* = 301)	Continuous (*n* = 297)
Eligibility criteria not fulfilled	4 (1.3)	6 (2.0)
Adverse events	1 (0.3)	6 (2.0)
Improvement/recovery	0	2 (0.7)
Lost to follow-up	6 (2.0)	7 (2.4)
Protocol non-compliance	2 (0.7)	2 (0.7)
Unsatisfied with symptom control	3 (1.0)	2 (0.7)
Other	3 (1.0)	4 (1.3)
Total	19 (6.3)	29 (9.8)^a^

^a^Difference versus on-demand treatment was not significant (*P* = 0.15)

For the primary variable (discontinuation because of unsatisfactory treatment), on-demand treatment was non-inferior to continuous treatment, as shown by the upper confidence limit (90 % CI) of the difference between on-demand and continuous treatment being less than 10 % in both the ITT and PP analyses (Table 3).

**Table 3:** Percentage of patients discontinuing due to unsatisfactory treatment in the intention-to-treat (ITT) and per-protocol (PP) populations

Treatment	Percentage of patients (*n*)	90 % confidence interval
ITT population		
Esomeprazole on-demand (*n* = 301)	6.3 (19)	
Esomeprazole continuous (*n* = 297)	9.8 (29)	
Difference (on-demand minus continuous)	−3.5	−7.1, 0.2
PP population		
Esomeprazole on-demand (*n* = 251)	1.2 (3)	
Esomeprazole continuous (*n* = 232)	0.4 (1)	
Difference (on-demand minus continuous)	0.8	−0.6, 2.1

The number of patients who discontinued prematurely from the study in each country was: Austria, 2 (3.3 %); France, 13 (8.5 %); Germany, 10 (6.8 %); South Africa, 14 (8.5 %); and Spain, 9 (12.0 %). When the primary variable was assessed by *H. pylori* status, a similar number of patients discontinued prematurely from the study whether they were *H. pylori*-positive (21 [8.2 %]) or not (27 [7.9 %]).

### Secondary variables

There was no statistically significant difference between the two treatment groups regarding the proportion of patients who responded that they were satisfied overall with the way their heartburn and regurgitation symptoms were being treated at 6 months (82.1 and 86.2 % for on-demand and continuous treatment, respectively; difference −4.1 % [95 % CI: −10.0 %, 1.7 %]). Similarly, there was no statistically significant difference in the proportion of patients satisfied with the way they were taking the treatment (81.7 and 82.8 % for on-demand and continuous treatment, respectively; difference −1.1 % [95 % CI: −7.2 %, 5.0 %]) and the effect of treatment (78.7 and 84.8 %, respectively; difference −6.1 % [95 % CI: −12.3 %, 0.1 %]).

Among patients with evaluable data (on-demand, *n =* 295; continuous, *n =* 286), mean drug usage over the 6-month study period was 0.41 (standard deviation [SD], 0.25) tablets (doses) per day in the on-demand treatment group and 0.91 (SD, 0.16) tablets per day in the continuous treatment group. Most patients took their medication in the morning (on-demand, 54.2 %; continuous, 72.5 %). In the on-demand treatment group, an analysis of reasons for taking the medication showed that more patients took their medication to soothe rather than to prevent symptoms (Table 4). However, most patients took medication to prevent symptoms at least once during the study.

**Table 4:** Reasons for drug intake in the esomeprazole on-demand treatment arm (intention-to-treat population, *n* = 301)

Have you taken your medicine to soothe or prevent symptoms?	Patients [*n* (%)]
To soothe	85 (28.2)
Mainly to soothe, sometimes to prevent	66 (21.9)
To both soothe and prevent	59 (19.6)
Mainly to prevent, sometimes to soothe	28 (9.3)
To prevent	15 (5.0)
Missing	48 (15.9)

The final endoscopy showed that most patients remained free of reflux esophagitis at the end of the 6-month treatment period; 15 patients (5 %), all in the on-demand treatment group, had endoscopic evidence of mucosal breaks at the end of the study (*P* < 0.0001 versus continuous treatment). Of these patients, 14 had LA grade A esophagitis and 1 had LA grade B.

**Fig. 3:**
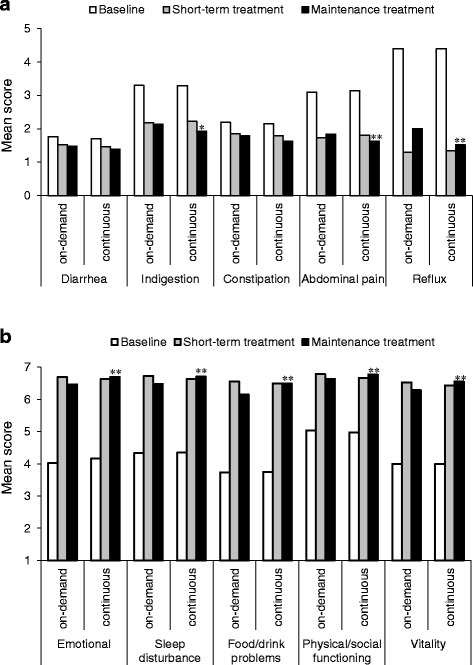
Mean (**a**) Gastrointestinal Symptom Rating Scale and (**b**) Quality of Life in Reflux and Dyspepsia questionnaire scores at baseline^a^, following 4 weeks’ initial (short-term) treatment with esomeprazole 20 mg once daily, and after maintenance treatment with either on-demand or continuous esomeprazole for 6 months (intention-to-treat population). ^a^Baseline corresponds to visit 1 (week −4). *P < 0.01 and **P ≤ 0.001 for the difference in the change in scores from baseline to the end of maintenance treatment for continuous versus on-demand treatment groups

From baseline to the end of the 4-week short-term treatment period (visit 1 to visit 2), large improvements in the GSRS Indigestion, Abdominal Pain and Reflux dimensions and in all QOLRAD dimensions were observed. During the randomized part of the study (maintenance therapy), further small improvements in scores were seen for the GSRS Indigestion and Abdominal pain dimensions in the continuous treatment group. Small improvements were also seen from baseline to the end of short-term treatment and to the end of maintenance treatment for the GSRS Diarrhea and Constipation dimensions. These improvements were evident in both the on-demand and continuous treatment groups. The mean GSRS scores at baseline, the end of short-term treatment and the end of maintenance treatment are shown in Fig. 3a. The estimated differences in the change in GSRS scores from baseline to the end of maintenance treatment between the continuous and on-demand treatment groups all favored continuous treatment: Diarrhea, 0.09 (*P* = 0.160); Indigestion, 0.25 (*P* = 0.002); Constipation, 0.14 (*P* = 0.050); Abdominal pain, 0.25 (*P* = 0.001); and Reflux, 0.54 (*P* < 0.001) (Fig. 3a). The estimated difference between the treatment groups in the Reflux dimension was of a magnitude considered to be clinically relevant. Mean QOLRAD scores at baseline and at the end of the short-term and maintenance treatment phases are shown in Fig. 3b. The estimated differences in the change from baseline to the end of maintenance treatment between the continuous and on-demand treatment groups were: Emotional, −0.28 (*P* < 0.001); Sleep disturbance, −0.28 (*P* < 0.001); Food/drink problems, −0.38 (*P* < 0.001); Physical/social functioning, −0.18 (*P* = 0.001); and Vitality, −0.31 (*P* < 0.001); all differences favored continuous treatment (Fig. 3b).

### Safety

Overall, the AE profile was similar between the two treatment groups, and the majority of AEs were mild or moderate in intensity. AEs were reported by 16.7 % of patients during the short-term treatment phase, and 36.2 and 35.4 % of patients during the on-demand and continuous maintenance phases, respectively. The most commonly reported AEs in the maintenance treatment phase were GI in nature (Table 5). There were no deaths during the study. In the maintenance phase, 1 patient in the on-demand treatment group and 7 patients in the continuous treatment group discontinued study treatment due to an AE (*P* = 0.07).

**Table 5:** Number of patients (%) with the most commonly reported adverse events in the maintenance treatment phase (≥2 % in any treatment group; safety population)

	Patients [*n* (%)]	
	On-demand (*n* = 301)	Continuous (*n* = 294^a^)
Flatulence	15 (5.0)	12 (4.1)
Abdominal pain	10 (3.3)	9 (3.1)
Diarrhea	6 (2.0)	9 (3.1)
Constipation	9 (3.0)	5 (1.7)
Viral infection	12 (4.0)	8 (2.7)
Headache	5 (1.7)	7 (2.4)
Respiratory infection	7 (2.3)	6 (2.0)
Gastroenteritis	7 (2.3)	9 (3.1)
Back pain	5 (1.7)	7 (2.4)
Arthralgia	2 (0.7)	6 (2.0)

^a^Three patients did not take any study drug and were therefore excluded from the safety population

SAEs were recorded for 4 patients in the on-demand treatment group and 11 patients in the continuous treatment group. With the exception of fracture, which occurred in 2 patients (1 humerus and 1 fibula), all SAEs occurred in 1 patient each (continuous treatment group: chest pain with dyspnea, renal pain, pulmonary embolism, aggravated angina pectoris, respiratory infection and hepatocellular damage, anemia, hernia [intervertebral disc], gastroenteritis and sinusitis; on-demand treatment group: meningitis, colon carcinoma, gastroenteritis and accidental injury with chest pain). However, none of the SAEs were considered by study investigators to be causally related to the study treatment. There were no laboratory findings that raised any safety concerns.

## Discussion

The results for the primary variable of this multinational study show that, following an initial 4-week symptom control phase with esomeprazole 20 mg once daily, 6 months’ on-demand maintenance treatment with esomeprazole 20 mg was non-inferior to continuous treatment in terms of patients’ willingness to continue treatment. The non-inferiority of on-demand versus continuous treatment was observed in both the ITT and PP populations, demonstrating that more serious protocol violations, such as the use of disallowed treatment to control reflux symptoms, did not appreciably alter the results for the primary variable. The results of the primary analysis were further supported by the finding that there was no statistically significant difference in the proportions of patients who were satisfied with the way of taking the drug, and with the effect of treatment. Indeed, although 18 and 14 % of patients in the on-demand and continuous treatment groups, respectively, were either indifferent to or dissatisfied with the way their heartburn and regurgitation symptoms were being treated, only 3 patients (1.0 %) in the on-demand treatment group and 2 patients (0.7 %) in the continuous treatment group discontinued due to unsatisfactory control of symptoms. This proportion is somewhat lower than was reported in an earlier 6-month study (*n* = 342) examining the efficacy of esomeprazole on-demand treatment in patients with NERD; in this study, 14 % of patients discontinued due to insufficient heartburn control (compared with 51 % in the placebo group) [12].

It is well recognized that rates of remission from endoscopic relapse in patients with GERD are directly related to the degree of acid suppression achieved during therapy [21], and PPIs are the most effective treatment option in this regard. Among the PPIs, esomeprazole provides sustained acid suppression [11] that has translated into higher rates of maintenance of reflux esophagitis healing compared with lansoprazole [22] and pantoprazole [23]. Esomeprazole 20 mg has also been approved in Europe for controlling symptoms in patients with NERD. Indeed, the efficacy of both esomeprazole 40 mg and 20 mg for the maintenance treatment of NERD has been demonstrated in placebo-controlled trials of continuous [24] and on-demand therapy [12, 20]. The on-demand use of PPIs, including esomeprazole, has also been compared with continuous use in several randomized clinical trials of patients with NERD or GERD [25–30]. Reviews have concluded that on-demand maintenance treatment with a PPI is an appropriate option for patients with mild reflux esophagitis and those with NERD [31–33], although some authors have questioned the efficacy of this approach for patients with healed reflux esophagitis [30]. Indeed, the results of an observational study suggest that an on-demand approach may more accurately match patient behavior, as some patients appear to use their treatment only as required for symptom control even when prescribed continuous therapy [34]. Despite extensive research in this area, to our knowledge, the present study is the first multinational study to investigate patient opinion of on-demand or continuous esomeprazole in patients with NERD who had responded to initial short-term treatment with a PPI.

Another potential advantage of on-demand treatment is that it is associated with lower medication use than continuous treatment. For example, a Japanese study examined the efficacy of maintenance treatment with omeprazole on-demand versus continuous in patients with NERD [35]. Over 24 weeks, mean study drug consumption ranged from 6.2 to 6.9 tablets per week in the continuous treatment group. In the on-demand treatment group, study drug consumption decreased over time, from 3.0 to 1.8 tablets per week [35]. In a further study, patients with NERD received esomeprazole on-demand or placebo for 6 months [12]. Mean intake of study medication was 0.34, indicating a 66 % reduction versus continuous once-daily intake [12]. In line with these findings, the present study showed that the use of study medication was reduced by 55 % with on-demand versus continuous treatment, yet the willingness of patients to continue therapy was similar to that achieved with continuous treatment (and with comparable HRQoL). Mean intake of study medication in the on-demand treatment group was higher than previously reported [12], although this may have been a result of patients taking their medication for prevention as well as relief of symptoms.

The reduction in PPI use with on-demand versus continuous treatment has important economic implications, as it is likely to correspond to a reduction in the cost associated with maintenance treatment [12]. Indeed, in one study that assessed 6-months’ maintenance treatment with esomeprazole 20 mg on-demand, esomeprazole 20 mg once daily or ranitidine 150 mg twice daily, direct medical costs in patients with GERD were €171.9, €221.6 and €248.8, respectively. The total costs associated with maintenance treatment were also lowest for on-demand esomeprazole (€221.5, €286.5 and €295.8, respectively) [32, 36]. Similarly, in another study, 6-months’ on-demand treatment with esomeprazole 20 mg incurred considerably lower direct medical costs than continuous treatment with omeprazole 20 mg once daily in GERD patients without esophagitis [37].

There is a known risk of relapse of reflux esophagitis during maintenance treatment, including on-demand therapy [31]. Not surprisingly, therefore, a small number of patients (*n* = 15), all of whom had been treated with esomeprazole 20 mg on-demand, had relapse with mucosal breaks at endoscopy after 6 months (the only objective observation in the present study), all of which were considered to be mild reflux esophagitis (14 with grade A and 1 with grade B, according to the LA classification). However, previous studies have found that patients may change between a non-erosive stage and reflux esophagitis [38, 39]. It is possible, therefore, that a small proportion of these 15 patients had mild reflux esophagitis that, at visit 1, was in remission, mediated by the limited use of PPI therapy that was permitted before inclusion in the study. Until recently, monitoring the course of NERD was very difficult; only with the advent of histological examination of biopsies from the Z-line has it been possible to monitor progression, regression and normalization during therapy [40–42]. Indeed, a previous study in patients with NERD reported that 3 and 6 months of therapy with omeprazole 40 mg led to complete recovery of dilation of intercellular spaces in more than 90 % of patients, and that in all cases this was associated with regression of heartburn [40]. Technological advances have also meant that NERD can now be diagnosed based on the absence of esophageal lesions on endoscopy, with the results of one study reporting that modern video gastroscopy was able to recognize 76.4 % of patients with NERD [43]. Combining histology and endoscopy could, therefore, improve the diagnosis and monitoring of disease progression during PPI treatment in patients with NERD.

Relative to on-demand treatment, continuous treatment was perhaps predictably associated with statistically significant improvements in single parameters of the GSRS and QOLRAD questionnaires. However, with the exception of the GSRS Reflux dimension, the differences in the change in GSRS and QOLRAD scores between the on-demand and continuous treatment groups were not deemed to be clinically relevant [44]. Moreover, patients in the on-demand treatment group took less medication than those randomized to continuous treatment, and the number of patients willing to continue treatment at 6 months was comparable between the two groups. Interestingly, despite the fact that 92 % of patients were assessed by the investigator as being ‘heartburn-free’ at the end of the 4-week short-term treatment period, patient-reported scores in the GSRS Reflux dimension were approximately 1.3 in both the on-demand and continuous treatment groups. This suggests some degree of disagreement between investigator and patient assessments. The difference, however, can probably be explained by the nature of the GSRS questionnaire; the GSRS Reflux dimension encompasses both heartburn and acid regurgitation, whereas in our study, investigator assessments (and subsequent inclusion in the maintenance phase) related exclusively to heartburn.

In this study, on-demand treatment could be taken to prevent symptoms, to soothe symptoms, or both. For example, patients may have chosen to take their medication to prevent symptoms in stressful situations, or before the occurrence of known reflux triggers. Although patients were asked whether they had taken medication to soothe or prevent symptoms, the exact reasons for usage of on-demand esomeprazole were not recorded. To further develop our understanding of patient behavior, more research into the reasons for taking on-demand medication is required.

The implications of *H. pylori* infection on management of GERD remains controversial [45]. For example, studies have shown that *H. pylori*-infected patients with GERD tend to have higher response rates to acid-suppressive treatment than *H. pylori-*negative patients. More than 40 % of patients in the present study were *H. pylori*-positive. This may have implications for the effectiveness of on-demand treatment, because *H. pylori* may be synergistic in preventing or treating reflux esophagitis when less than optimal acid suppression is used [46, 47]. Nonetheless, the primary variable was unaffected by *H. pylori* status in the present study.

Limitations of this study include its open-label nature and the fact that patients underwent endoscopy at study end and upon discontinuation, but not at regular intervals during the study. In addition, the study only included NERD patients who had complete resolution of heartburn symptoms following initial treatment with esomeprazole; therefore, it is possible that results may have been less favorable in patients whose response to short-term treatment was not complete. Strengths of the study, on the other hand, include the multinational nature of the study population, the use of validated instruments to assess symptoms (GSRS) and quality of life (QOLRAD), and the use of three different measures of patient opinion about the impact of on-demand or continuous treatment (discontinuation due to unsatisfactory treatment, standardized treatment satisfaction questionnaire and the quality of life questionnaire).

## Conclusions

Using the measure ‘willingness to continue treatment’, 6 months of on-demand esomeprazole maintenance treatment was non-inferior to continuous maintenance treatment in patients with NERD who had achieved complete symptom resolution following 4 weeks of treatment with esomeprazole. Although continuous treatment gave significantly better symptom control than on-demand treatment, as measured by the GSRS questionnaire, only the difference in the Reflux dimension reached a magnitude that was clinically relevant. As may have been expected, medication intake was lower with on-demand treatment, with obvious potential benefits in terms of cost of treatment and convenience to patients.

## Abbreviations

AE: adverse event
CI: confidence interval
GERD: gastroesophageal reflux disease
GI: gastrointestinal
GSRS: Gastrointestinal Symptom Rating Scale
HRQoL: health-related quality of life
ITT: intention to treat
NERD: non-erosive reflux disease
PP: per protocol
PPI: proton pump inhibitor
QOLRAD: Quality of Life in Reflux and Dyspepsia
SD: standard deviation
